# HIV testing in a South African Emergency Department: A missed opportunity

**DOI:** 10.1371/journal.pone.0193858

**Published:** 2018-03-13

**Authors:** Bhakti Hansoti, David Stead, Andy Parrish, Steven J. Reynolds, Andrew D. Redd, Madeleine M. Whalen, Nomzamo Mvandaba, Thomas C. Quinn

**Affiliations:** 1 Department of Emergency Medicine, Johns Hopkins University School of Medicine, Baltimore, MD, United States of America; 2 Department of Medicine, Faculty of Health Sciences, Walter Sisulu University, Mthatha, South Africa; 3 Department of Internal Medicine, Frere and Cecilia Makiwane Hospitals, East London, Eastern Cape, South Africa; 4 Department of Medicine, Johns Hopkins University School of Medicine, Baltimore, MD, United States of America; 5 Division of Intramural Research, National Institute of Allergy and Infectious Diseases, NIH, Bethesda, MD, United States of America; Yale University Yale School of Public Health, UNITED STATES

## Abstract

**Background:**

South Africa has the largest HIV epidemic in the world, with 19% of the global number of people living with HIV, 15% of new infections and 11% of AIDS-related deaths. Even though HIV testing is mandated in all hospital-based facilities in South Africa (SA), it is rarely implemented in the Emergency Department (ED). The ED provides episodic care to large volumes of undifferentiated who present with unplanned injury or illness. Thus, the ED may provide an opportunity to capture patients with undiagnosed HIV infection missed by clinic-based screening programs.

**Methods and findings:**

In this prospective exploratory study, we implemented the National South African HIV testing guidelines (counselor initiated non-targeted universal screening with rapid point of care testing) for 24-hours a day at Frere Hospital in the Eastern Cape from September 1st to November 30th, 2016. The purpose of our study was to quantify the burden of undiagnosed HIV infection in a South African ED setting. Furthermore, we sought to evaluate the effectiveness of the nationally recommended HIV testing strategy in the ED. All patients who presented for care in the ED during the study period, and who were clinically stable and fully conscious, were eligible to be approached by HIV counseling and testing (HCT) staff to receive a rapid point-of-care HIV test. A total of 2355 of the 9583 (24.6%) patients who presented to the ED for care during the study period were approached by the HCT staff, of whom 1714 (72.8%) accepted HIV testing. There was a high uptake of HIV testing (78.6%) among a predominantly male (58%) patient group who mostly presented with traumatic injuries (70.8%). Four hundred (21.6%) patients were HIV positive, including 115 (6.2%) with newly diagnosed HIV infection. The overall prevalence of HIV infection was twice as high in females (29.8%) compared to males (15.4%). Both sexes had a similar prevalence of newly diagnosed HIV infection (6.0% for all females and 6.4% for all males) in the ED.

**Conclusions:**

Overall there was high HIV testing acceptance by ED patients. A non-targeted testing approached revealed a high HIV prevalence with a significant burden of undiagnosed HIV infection in the ED. Unfortunately, a counselor-driven HIV testing approach fell short of meeting the testing needs in this setting, with over 75% of ED patients not approached by HCT staff.

## Introduction

HIV testing is the critical first step to meeting the WHO global strategy goal of having 90% of HIV-infected individuals tested, 90% of those on anti-retroviral treatment (ART), and 90% of those virally suppressed (90-90-90).[1] Early detection of undiagnosed HIV with effective linkage to care and ART uptake is also widely recognized to extend life expectancy, improve quality of life, and reduce HIV transmission.[2] Strengthening HIV testing programs will be vital to achieving the 90-90-90 target.

In 2016, South Africa had 270,000 new HIV infections, 110,000 AIDS-related deaths and over 7.1 million people living with HIV.[3, 4] The bulk of HIV testing resources, in this setting, have focused on primary health care centers and antenatal clinics with almost universal availability of testing and treatment services for pregnant women.[3, 5–7] A 2012 South African National household survey found that only 65.5% of adults 15 years and older had ever been tested for HIV, with only 59% of males having ever been tested compared to 71.5% of females.[3] This gender discrepancy in testing in part leads to only 43% of HIV positive males being engaged in care compared to 60% of females.[8]

Recent innovations in HIV testing often focus on difficult to access and high-risk populations such as prisoners, commercial sex workers, and injecting drug users. [9–11] One segment of the population who are unlikely to interact with the healthcare system and not frequently captured by targeted interventions for special populations are young men.[12, 13] The Emergency Department (ED) is an episodic care center that provides care to large volumes of patients.[14] Compared to other available health care access points, ED populations are typically younger and have a higher prevalence of substance abuse and mental health disorders. [15] In North America, researchers have found ED populations to have a higher prevalence of HIV infection compared to antenatal clinics and other ambulatory care settings.[16–18] Importantly, 20–27% of these HIV positive patients in the ED were previously unaware of their HIV status.[19–22] In 2006, the United States Centers for Disease Control and Prevention (CDC) explicitly recognized the critical roles of EDs in the national HIV strategy after programs reported high rates of detection and linkage to care from this venue. [19, 23–25] Several studies in Low and Middle-Income Countries (LMICs) have also demonstrated high HIV prevalence in the ED population (23% in Kenya, 26% in Malawi and 43% in Uganda).[26–28] HIV testing in South African EDs may provide an opportunity to capture patients who are currently missed by existing testing programs.

Despite the 2015 National South African HIV testing guidelines mandating universal testing in all healthcare facilities, ED-based HIV testing is not routinely implemented in SA.[29, 30] Frequently cited barriers to offering HIV testing in the ED include limited time, inadequate resources, and concerns regarding the provision of follow-up care.[31, 32] In this exploratory study, we implemented the nationally recommended counselor-initiated point-of-care (POC) HCT program. This strategy mandates the use of extensive pre-and post-test counseling and HIV education. The purpose of this prospective study was to quantify the burden of undiagnosed HIV infection in a South African ED setting. Furthermore, we sought to evaluate the effectiveness of the nationally recommended testing model at HCT service delivery in the ED.

## Methods

### Study design and setting

This prospective study was conducted at Frere Hospital in East London, SA from September 1^st^ to November 30^th^, 2016. Frere Hospital is an urban tertiary care center that provides 24-hour emergency medical and trauma care to the greater East London area, as well as for referrals from regional and district hospitals up to 180km away. The ED has approximately 35 beds and two doctors on staff at all times. There is no Electronic Health Record (EHR) or patient tracking system. Patients wait in line and are seen in order of presentation unless they are critically ill.

### HIV testing intervention

During the study period, HIV testing was implemented and conducted per the recommendations and protocols in the 2015 National HCT guidelines, as was required by the local ethics review board. [29] The core recommendations of the national guidelines mandate non-targeted universal screening with an opt-in testing approach; requiring informed consent with extensive pre-and-post-test counseling in a confidential private setting.

HIV testing in SA is often conducted by lay counselors (i.e., non-medical persons). All HCT staff had been previously trained in rapid POC HCT and had been previously employed as lay HCT providers in the neighboring area. Staff were required to be fluent in English, Xhosa and/or Afrikaans and have a minimum 12^th^-grade education. HCT staff received remuneration in line with current salaries for lay HCT providers (approximately 150 ZAR ($10)/day). Before starting the study, all staff were required to complete Good Clinical Practice (GCP) and data collection training, as well as an HCT refresher course provided by Department of Health certified trainers from the antenatal clinic at Frere Hospital. Staff worked in 12-hour shifts to cover the ED 24 hours a day. At all times two HCT staff were available to conduct HIV testing.

The HIV testing algorithm implemented used rapid POC blood tests approved by the South African Department of Health. Blood samples were obtained using a finger-prick. All patients were first tested with the Advanced Quality^TM^ Rapid Anti-HIV 1& 2 test (InTec Products, Inc, Xiamen, China). Non-reactive specimens were considered HIV-negative and reported as such. All reactive specimens were confirmed using an ABON™ HIV 1/2/O Tri-line HIV Rapid Test (ABON Biopharm, Hangzhou, China). The Advanced testing kit provided results within 10 minutes and the ABON kit within 15 minutes. The total time to complete HCT for an HIV negative patient was between 30–40 minutes, and for an HIV positive patient was up to 60 minutes.

As per the 2015 National Testing Guidelines, patients with two reactive rapid tests were considered to be HIV infected, and no further confirmatory testing was done. Patients were provided with their HIV results during the ED stay (usually within twenty minutes of collecting the specimen). HIV positive patients were provided with a letter stating their results, as is standard of care, to facilitate referral to a local anti-retroviral (ARV) clinic. Specimens that were reactive on the first assay but non-reactive on the second were subject to an ELISA laboratory test and recorded as discordant (no discordant results were generated during this study). Testing kits were stored in a cool room, with daily room temperature logs. Each kit was first quality control-checked using known reactive and non-reactive controls.

### Recruitment and enrolment

All patients who presented for care in the ED during the study period and were over the age of 18 years, fully conscious, and clinically stable were eligible to be approached by HCT staff. As implemented, the HIV counselors were instructed to approach all patients who were eligible as soon as they completed the triage process. There was a private waiting area after the triage station where HCT providers could approach patients. Staff were instructed to try and capture all patients who presented for care to the ED.

### Outcomes and data collection

The primary outcome measure was the proportion of patients with newly diagnosed HIV infection among those who were tested by the HCT staff in the ED. Secondary outcome measures included patient characteristics such as age, sex, presenting complaint, South African Triage Scale (SATS) score, disposition and previous diagnosis of HIV infection. Presenting complaint was recorded as free text and coded using Medical Dictionary for Regulatory Activities (MedDRA). The severity of illness was quantified using SATS (a locally implemented triage score), as is standard of care.[33] Data were prospectively collected on case report forms by HCT staff, who also noted whether or not subjects consented to a rapid POC HIV test, and their POC HIV test results. Data forms were scanned and entered using intelligent character recognition (ICR) DataFax software (DataFax, Clinical DataFax Systems Inc., Hamilton, Ontario, Canada) and centrally double-verified by independent data technicians.

### Sample size and data analysis

Based on the recent survey data from the 2012 South African National HIV prevalence study, HIV prevalence is estimated at 12.2%.[3] Our study aimed to recruit a sample size of 620 patients. This would allow us to detect a difference of greater than 5% from the baseline estimate of 12.2%, with a 95% confidence interval and power of 0.8. Data were analyzed using a descriptive statistical approach using STATA v.12 (StataCorp, LLC, Texas). The overall HIV prevalence was calculated by including patients with a known history of HIV infection and HIV-positive patients newly identified by HCT in the ED. The uptake of HCT was quantified as the proportion of patients who accepted HCT relative to those offered HCT. Univariate comparisons of patient characteristics by HIV status were conducted using chi-squared tests. We employed generalized linear models and logistic regression to assess the association between sex, presenting complaint, time of testing, symptomatology and HIV status.

### Ethical considerations

This study was approved by the Johns Hopkins University School of Medicine Institutional Review Board, the University of Cape Town Human Research Ethics Committee, and the Walter Sisulu University Human Research Ethics Committee. Written consent was obtained from all participants who enrolled in the study and was required for both the collection of demographic data and POC HIV testing results. Approval was not given to collect de-identified data on patients that presented to the ED but did not participate in the study, i.e., did not consent to an HIV test.

## Results

**Fig 1:**
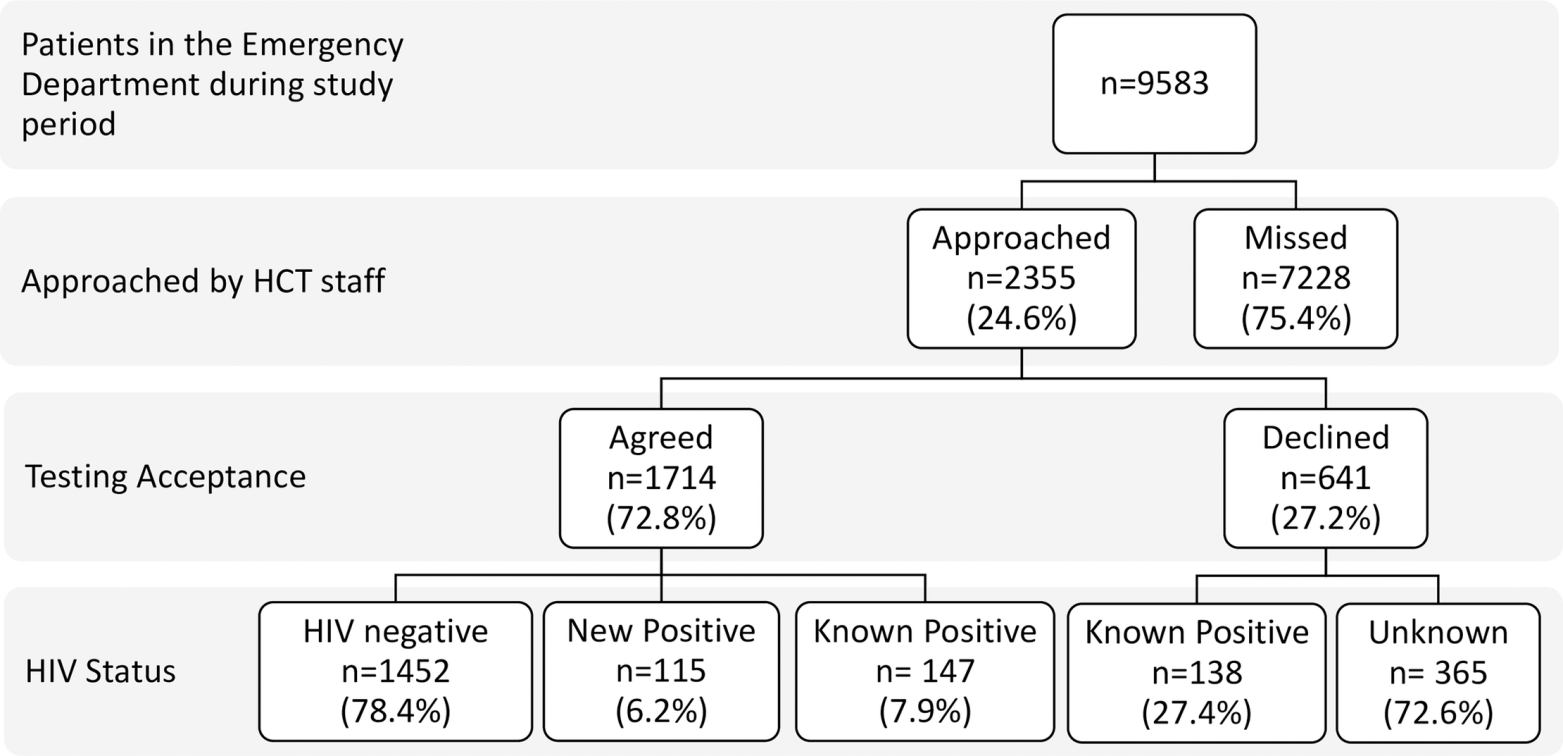
Patient enrollment and testing acceptance overview.

A total of 2355 of the 9583 (24.6%) patients that presented to the ED for care during the study period were approached by the HCT staff (Fig 1).

Of the 2355 patients approached by the HCT staff, 1714 patients (72.8%) accepted testing, and 641 patients (27.2%) declined to test. Of those who declined HIV testing, 138 patients (27.4%) stated that they had a known diagnosis of HIV. In this study, we could not collect any further data on the reasons for refusing to test. Of the 1714 rapid POC HIV tests performed, 262 tests were positive and 1452 tests negative. Of the 262 patients who had a positive HIV test in the ED 115 patients stated that this was a new HIV diagnosis (Fig 1). One hundred and forty-seven patients with a known HIV diagnosis re-tested in the ED. When patients were informally asked why they wanted a repeat test, many stated that they want to see if they still had the disease or that they didn’t believe their previous test results.

Baseline demographics were collected on all patients approached by HCT staff (Table 1). Compared to those who accepted a rapid POC test, a higher percentage of those who declined testing were male (308, 61.2%), and presented for the evaluation of a traumatic injury (385, 76.5%). There were no trends when evaluating the mean age or severity of illness. However, a slightly higher proportion of those who accepted testing was likely to be admitted (383, 20.7%) compared to those who declined to test (78, 15.5%).

**Table 1:** Characteristics of all participants stratified by testing acceptance (N = 2355).

	*Declined Testing (n = 641)*	*Accepte Testing (n = 1714)*	*Total (n = 2355)*
Age, mean in years	36.8	36.9	36.9
(95% CI)	(35.6–38.1)	(36.3–37.6)	(26.4–37.5)
Sex			
Female	195 (38.8%)	799 (43.1%)	994 (42.2%)
Male	308 (61.2%)	1053 (56.9%)	1361 (57.8%)
Presenting Complaint			
Medical	118 (23.5%)	576 (31.1%)	694 (29.5%)
Trauma	385 (76.5%)	1276 (68.9%)	1661 (70.5%)
SATS*			
Emergency	1 (0.2%)	7 (0.4%)	8 (0.3%)
Very Urgent	71 (14.2%)	331 (18.0%)	402 (17.0%)
Urgent	142 (28.2%)	590 (31.8%)	732 (31.1%)
Routine	284 (56.5%)	893 (48.3%)	1177 (50.0%)
Deceased	1 (0.2%)	0 (0%)	1 (0.1%)
Unassigned	4 (0.8%)	27 (1.5%)	31 (1.3%)
Disposition			
Death	0	3 (0.2%)	3 (0.1%)
Admission	78 (15.5%)	383 (20.7%)	461 (19.6%)
Emergent Surgery	3 (0.6%)	3 (0.2%)	6 (0.3%)
Transfer	14 (2.8%)	68 (3.7%)	82 (3.5%)
Discharge	394 (78.3%)	1341 (72.4%)	1735 (73.7%)
Absconded	5 (1.0%)	25 (1.3%)	30 (1.3%)
Unassigned	9 (1.8%)		

Note: Values are number (% rounded to 1 decimal place) unless otherwise noted

* South African Triage Score (SATS)

HIV status was determined in 1852 patients (including all the patients tested in the ED (n = 1714) and those who declined testing but were known positive (n = 138)) (Table 2). Of these 1852 patients, 400 patients were HIV positive (by POC HIV test or by history) giving an HIV prevalence of 21.6%. Of these 400, 115 (28.8%) reported being previously unaware of their status giving a prevalence of newly diagnosed HIV infection in the ED of 6.2%. The mean age of the newly diagnosed HIV positive patients was significantly lower (33.9 years, 95% CI 31.9–35.9), compared to HIV negative patients (37.1 years, 95% CI 36.3–37.9), and the known HIV positive patients (37.4 years, 95% CI 36.1–38.9).

**Table 2:** Characteristics of participants who consented to HIV testing or knew their HIV status, stratified by HIV status (N = 1852).

	HIV negative	Known HIV positive	New HIV positive	P-value
	(n = 1452)	(n = 285)	(n = 115)	
	(78.4%)	(15.4%)	(6.2%)	
Age, mean in years (95% CI)	37.1 (36.3–37.9)	37.4 (36.1–38.6)	33.9 (31.9–35.9)	ANOVA F(2) = 1.85 P = 0.1359
Sex				
Female	561 (38.6%)	190 (66.7%)	48 (41.7%)	Chi2(2) = 76.4072 P = <0.001
Male	891 (61.4%)	95 (33.3%)	67 (58.3%)	
Presenting Compliant				
Medical	412 (28.4%)	111 (39.0%)	53 (46.1%)	Chi2(3) = 25.2773 P<0.001
Trauma	1040 (71.6%)	174 (61.0%)	62 (53.9%)	
SATS*				
Emergency	6 (0.4%)	0 (0.0%)	1 (0.9%)	Chi2(8) = 8.9342 P = 0.348
Very Urgent	249 (17.2%)	58 (20.4%)	27 (24.5%)	
Urgent	460 (31.7%)	89 (31.2%)	41 (35.7%)	
Routine	715 (49.2%)	135 (47.4%)	44 (38.3%)	
Unassigned	22 (154%)	3 (1.1%)	2 (1.7%)	

Note: Values are number (% rounded to 1 decimal place) unless otherwise noted

* South African Triage Score (SATS)

**Fig 2:**
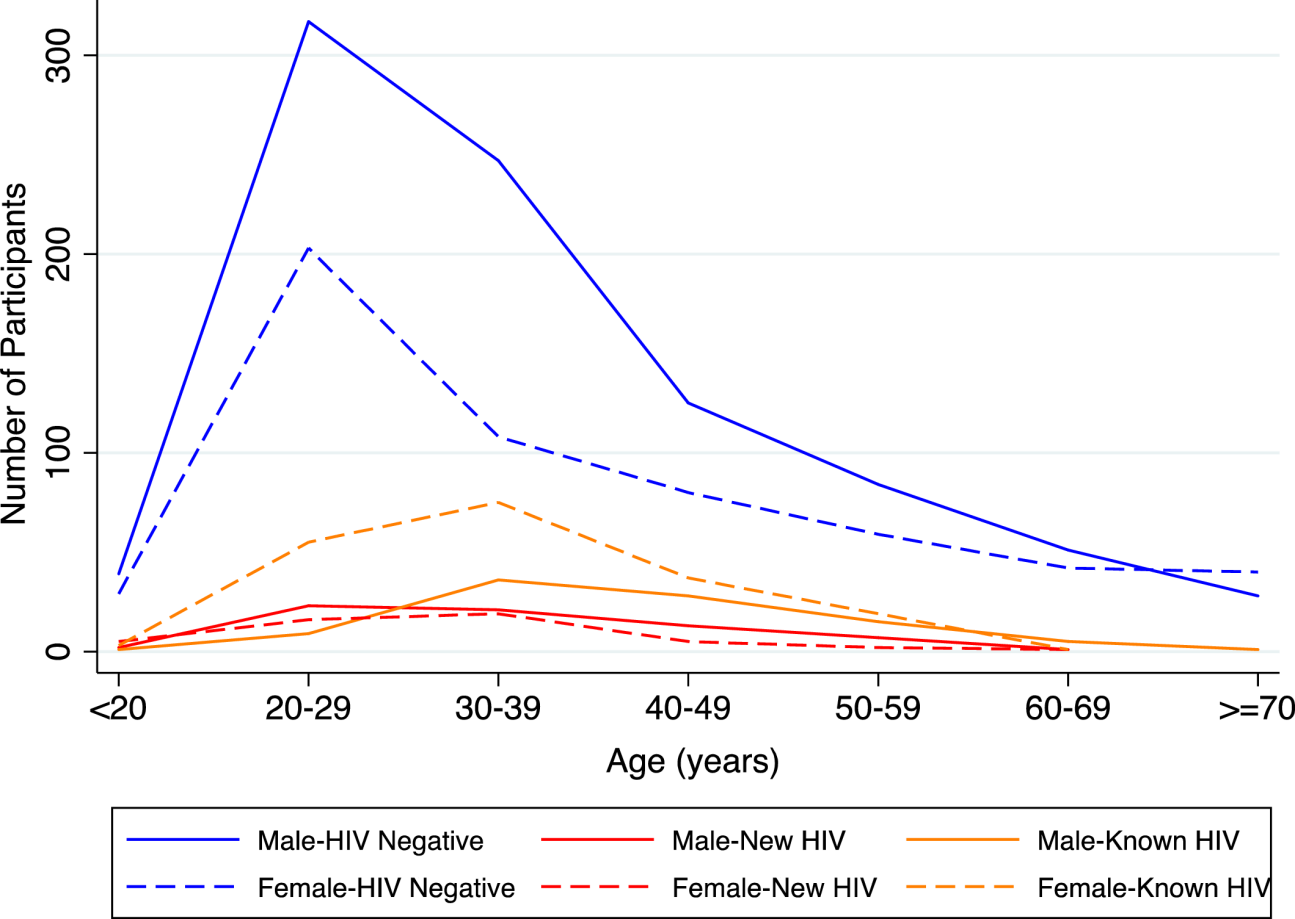
Stratification of HIV status by age and gender.

The overall burden of newly diagnosed HIV infection in the ED was similar in females (6.0%) and males (6.4%). However, the prevalence of known HIV infection in females (23.8%) was more than twice that in males (9.0%). This led to an overall prevalence of HIV infection for females of 29.8% and 15.4% for males (Table 2). Fig 2 provides stratification of HIV status by age and gender and illustrates that the peak prevalence of newly diagnosed HIV infection occurs in males at a younger age than their female counterparts. Female HIV positive patients were more likely to be aware of their HIV status (79.8%, 190/238) compared to their male counterparts (58.6%, 95/162).

A significantly higher proportion of patients presented with traumatic injuries in all three groups (HIV negative, Known HIV positive, and New HIV positive) (Table 2). There was no difference in the severity of illness among patients in all three groups (Table 2). Seven patients who participated in the study were initially assigned a SATS triage level of emergency; of these, two patients had a seizure on arrival, four were initially hypoglycemic, and one had a tachycardia secondary to pain. All of these patients consented to participate once their initial complaints had been resolved.

The critical focus of an HIV testing intervention is to capture those patients with a new diagnosis of HIV infection (n = 115) among those whose status is unknown (n = 1567). Overall the relative risk of a new diagnosis of HIV infection was not significantly impacted by gender, time of testing or symptomatology (Table 3). Furthermore, the majority of patients with a new diagnosis of HIV infection had no symptoms of opportunistic infections (112, 97.4%) compared to only three (2.6%) who had one or more of fevers, weight loss, difficulty breathing or a cough (Table 3). Thus, this analysis did not identify any patient characteristics which could be utilized in targeted screening algorithm.

**Table 3:** Relative likelihood of a new diagnosis of HIV infection among all patients with previously unknown status.

	RR of New Diagnosis of HIV infection (n = 115) among all of those previously unknown (n = 1567)		
	n	Relative Risk*	95% CI
Sex			
Female	48	ref	
Male	67	0.95	0.81–1.11
Presenting Complaint			
Medical	53	ref	
Trauma	62	0.49	0.35–0.70
Time of Testing			
Between 9a-5p	51	ref	
Out of Hours	64	0.99	0.69–1.41
Symptomatic			
No symptoms	112	ref	
One of more of fevers, weight loss, difficulty breathing or a cough	3	2.77	0.99–7.75

*Note: Relative Risk and 95% Confidence Interval are given to 2 decimal places unless otherwise noted

In Table 4 we present a logistic regression analysis comparing patients with a new diagnosis of HIV infection (n = 115) compared to all HIV positive individuals in this study (n = 400). This analysis identified that among HIV positive patients in the ED, HIV positive males were more likely to be unaware of their status compared to HIV positive females (1.75, 95% CI 1.39–2.19). However, patients with newly diagnosed HIV infection did not demonstrate any other significant differences compared to known HIV positive counterparts.

**Table 4:** Relative likelihood of new diagnosis of HIV infection among all HIV positive patients.

	*RR of New Diagnosis of HIV infection (n = 115) among all HIV positive patients (n = 400)*		
	*n*	Relative likelihood*	*95% CI*
Sex			
Female	48	ref	
Male	67	1.75	1.39-.2.19
Presenting Compliant			
Medical	53	ref	
Trauma	62	0.81	0.60–1.11
Time of Testing			
Between 9a-5p	51	ref	
Out of Hours	64	0.88	0.65–1.20
Symptomatic			
No symptoms	112	ref	
One or more of fevers, weight loss, difficulty breathing or a cough	3	1.04	0.40–2.73

*Note: Relative Risk and 95% Confidence Interval are given to 2 decimal places unless otherwise noted

## Discussion

This exploratory study demonstrates a high burden of HIV infection in a South African ED setting. The prevalence of HIV infection in our ED population (21.6%) is considerably higher than the reported estimates for the Eastern Cape province (12.2%), and the local district (13.6%).[3] This high HIV prevalence is in keeping with other African ED studies from Kenya (23%), Malawi (26%), and Uganda (43%) [26–28]. The majority of patients in our study were male (1361, 57.8%), presented with traumatic injuries (1661, 70.5%) and under the age of 40 years. This is consistent with other reports on ED populations in LMICs, a systematic review of 192 emergency care facilities in 59 LMICs demonstrated that the majority of patients are male (median 55.7%) and young (median 35 years). [14] This is in contrast to the majority of testing reports from mobile clinics and other ambulatory care facilities that report a female predominance in their testing populations.[34–36] Thus expanding HCT programs to the ED may be ideally suited to access young male patients. The burden of newly diagnosed HIV infection in our study was 6.2%, the majority of these patients came to the ED for evaluation of traumatic injuries (58.3%), and their new diagnosis of HIV was an incidental finding.

The overall HIV prevalence in females was almost twice that of males in our study. This rate is higher when compared to the national HIV figures where females outnumber males by a factor of 1.45 (14.4% compared to 9.9%) and thus may reflect higher population burden of HIV among females in the area compared to the national average. [3] We also identified that female HIV patients in our cohort were also more likely to be aware of their HIV status. This trend is consistent with the national surveys and likely reflects in part the greater accessibility of HCT to women through routine opt-out testing programs in antenatal clinics (i.e., where every patient is tested without the need for extensive counseling or informed consent), as well as primary health care centers.[37–39]

Over 78% of patients accepted an HIV test when offered, demonstrating that patient refusal was not a barrier to the implementing an ED-based testing program. Acceptance of testing in our study was considerably higher than the testing uptake reported in a South African out-patient setting (49%), and the National household survey (67.5%). (5, 49) Although men were more likely to refuse to test than women, our study still managed to capture a majority of male participants for HCT (56.9%). One of the challenges and arguments against providing routine HCT in the ED is the high stress, sometimes chaotic environment, with critically ill patients requiring urgent care. Documenting an HIV diagnosis in this setting is not thought of as a priority.[31, 40, 41] Our study revealed a high rate of POC test acceptance across the SATS severity grades, indicating that critical illness was not a significant obstacle in practice to patients. Interestingly we also found that patients who reported a known diagnosis of HIV infection requested repeat testing in the ED. When patients were informally asked why they wanted a repeat test, many stated that they want to see if they still had the disease or that they did not believe their previous test results. The repeat testing phenomena may speak to the low health literacy in the region or the inherent mistrust in testing programs given that over 50% of patients with a known HIV diagnosis requested a repeat POC test in the ED when offered. The high volume of known HIV positive patients that present to the ED may be an opportunity to provide linkage to care or ART initiation services in a population that is likely to have poor engagement.

Unfortunately, despite having two HCT counselors providing testing services 24 hours a day, we were only able to approach a quarter of the patients who presented for care. In part, we believe that this poor capture was due to time-consuming nature of the HIV testing process that was implemented. The current testing guideline, require extensive pre- and post-test counseling and can take 30 to 40 minutes to complete (which almost doubles for HIV positive patients due to the need for confirmatory testing). Some studies have also cited that long wait times may contribute to test refusal.[34]

The number needed to test for each new positive case was 16:1 yielding on average one new HIV case per day. Some may suggest targeted testing in a setting where HCT resources are limited. However, our study also demonstrated that a targeted testing approach by gender, presenting complaint, or the presence of TB symptoms, would miss many of the patients we captured with undiagnosed HIV infection and underlines the importance of a universal HIV screening approach in the ED. [2, 37, 39, 42–46]

The implemented strategy required an eight-person HCT team to provide 24-hour testing services, costing on average 600 ZAR ($40)/day. This human resource burden may be difficult to maintain in a real-world setting where human resources are limited. Also, because a significant proportion of HIV testing (55.3%) was conducted outside of regular business hours (defined as 9 am to 5 pm), a traditional facility-based HCT strategy would have missed this population. Studies have identified that lay counselor programs lack standardized training and supervision and that competing administrative/auxiliary duties may lead to reduced service delivery.[30] A more integrated approach that places the impetus for testing on existing healthcare providers in the ED may be necessary to implement a sustainable and effective HIV testing program. Future endeavors should consider an implementation science approach to developing innovative HIV testing programs within this setting. [2, 47, 48]

A significant limitation of this study is that only a quarter of the ED population was approached by HCT staff. Due to the lack of an EHR/patient-tracking system, we cannot capture information on those who were missed. Thus, we were unable to perform a sensitivity analysis on the patients that were missed. It is possible that there is a bias in the patients included in this study, such as they were easier to approach, more likely to speak the same language as the HCT staff, and presented at times when the patient volume in the ED was lower. This may limit the generalizability of the study to the overall population attending the ED and in the community. Nevertheless, our study did reveal a relatively high prevalence of HIV infection among those who were tested. Future studies will need to capture the whole of the ED population to present a more accurate assessment of the true burden of HIV in this setting. Furthermore, information on linkage to care for these patients is not available, which would have been necessary to assess the downstream impact of the testing program on the 90-90-90 targets.

## Conclusion

This evaluation of the nationally recommended HCT strategy in a South African ED demonstrated a high acceptance of HIV testing in the ED, with 6.2% of those being tested having a new diagnosis of HIV infection. There were no significant demographic or clinical risks for HIV infection identified, which argues for routine universal HIV testing in the ED. Further innovation and implementation research is required to develop a sustainable testing strategy integrated into the clinical environment so that service delivery in the ED can be improved.
